# Spatio-Temporal Analysis of Drought Variability Using CWSI in the Koshi River Basin (KRB)

**DOI:** 10.3390/ijerph16173100

**Published:** 2019-08-26

**Authors:** Han Wu, Donghong Xiong, Bintao Liu, Su Zhang, Yong Yuan, Yiping Fang, Chhabi Lal Chidi, Nirmal Mani Dahal

**Affiliations:** 1 Institute of Mountain Hazards and Environment, Chinese Academy of Sciences, Chengdu 610041, China; 2 University of Chinese Academy of Sciences, Beijing 100049, China; 3 Sino-Nepal Joint Research Centre for Geography, IMHE-TU-YNU, Kathmandu 44600, Nepal; 4 Branch of Mountain Sciences, Kathmandu Center for Research and Education, CAS-TU, Kathmandu 44600, Nepal; 5 Central Department of Geography, Tribhuvan University, Kathmandu 44600, Nepal

**Keywords:** drought, MODIS, CWSI, Koshi river basin

## Abstract

Drought is one of the most frequent meteorological disasters, and has exerted significant impacts on the livelihoods and economy of the Koshi River Basin (KRB). In this study, we assessed drought patterns using the Crop Water Shortage Index (CWSI) based on the MOD16 product for the period between 2000 and 2014. The results revealed that the CWSI based on the MOD16 product can be act as an indicator to monitor the characteristics of the drought. Significant spatial heterogeneity of drought was observed in the basin, with higher CWSI values downstream and upstream than in the midstream. The midstream of the KRB was dominated by light drought, moderate drought occurred in the upstream, and the downstream was characterized by severe drought. The monthly CWSI during one year in KRB showed the higher CWSI between March to May (pre-monsoon) and October to December (post-monsoon) rather than June to September (monsoon), and the highest was observed in the month of April, suggesting that precipitation plays the most important role in the mitigation of CWSI. Additionally, the downstream and midstream showed a higher variation of drought compared to the upstream in the basin. This research indicates that the downstream suffered severe drought due to seasonal water shortages, especially during the pre-monsoon, and water-related infrastructure should be implemented to mitigate losses caused by drought.

## 1. Introduction

Drought is one of the most devastating, stochastic and recurring natural hazards around the world, and has exerted negative impacts on agricultural production, groundwater supply, modern industrial production and electricity production [1,2,3,4,5]. According to statistics from the International Disaster Database (EM-DAT), global annual loss caused by drought was around 221 billion dollars from 1960 to 2016. In addition, the estimation of IPCC showed that climate change is expected to cause more frequent and intense drought periods, which may even occur in regions where an increase in precipitation are expected [6,7,8]. Therefore, it is necessary to monitor drought characteristics with the target of protecting the eco-environment and reducing unnecessary loss of life and money.

In general, based on different types of water deficits, droughts are generally categorized into four major types: meteorological droughts, agricultural droughts, hydrological droughts and socio-economical droughts [9,10]. Among these four types, agricultural drought is recognized as a recurring, non-structured natural disaster which plays a major role in the economy of agrarian countries, such as India and Nepal, where more than 68% of people are dependent on agriculture production [11]. The occurrence of drought makes the land incapable of cultivation throughout the year, exerting a huge negative impact on human beings and livestock populations, as well as biomass potential and plant species [11]. In recent years, many drought indices have been developed to quantify and monitor different drought events [12]. Numerous indices, including the Palmer Drought Severity Index (PDSI) [13], Standardized Precipitation Index (SPI) [14], and the Standardized Precipitation Evapotranspiration Index (SPEI) [15,16], have been widely used in many countries around the world. Although the above mentioned indices have higher authenticity, it is difficult to reflect the status of intensive drought due to the limitation of the density of gauge networks [17]. Since the 1970s, many researchers have used wide-cover, high-resolution satellite observation data to monitor drought since remote sensing has the potential for providing a spatial context and a synoptic view of the land for measuring drought impacts [18]. Drought indices derived from remote sensing-based measurements are widely used to monitor agricultural drought conditions as well [19]. Compared with conventional meteorological or hydrological measurements based on observation stations, the remote sensing (RS)-based drought indices is characterized by macroscopic, economic, dynamic and timely styles, which has also been regarded as an important complement to conventional agricultural hazards monitor [19].

Presently, many RS-based drought indices, e.g., the Anomaly Vegetation Index (AVI) [20], Vegetation Condition Index (VCI) [21], Apparent Thermal Inertia (ATI) [22], Crop Water Stress Index (CWSI) [23], and Temperature Vegetation Dryness Index (TVDI) [24] are used to monitor drought on a regional scale. Of these indices, CWSI has a high measurement precision as well as a clear physical meaning, which does not require the study area to have all land cover types [25]. Therefore, many studies were conducted on drought based on CWSI to monitor drought dynamics in many regions [26,27,28].

The Koshi River Basin (KRB) is a transboundary basin flowing through China, Nepal and India, with a total area of 69,300 km^2^. Climate Change in the KRB is severely affecting water resources, agriculture, food security, and local livelihoods [29]. Drought is the greatest natural hazard in Nepal with a total of 11 times of large-scale drought between 1905 and 1975 [30]. However, a few studies were conducted to show the drought characteristic to guide the agricultural production and mitigate the hazard loss. Consequently, it is highly important to understand the drought pattern in order to assist decision makers in preparing appropriate strategies for the changing conditions therein. Unfortunately, few stations are established in mountainous areas of the Central Himalaya, and usually they lack long-term records [31]. In response to the abovementioned limitations, satellite remote sensing provides spatially contiguous high-accuracy and high-resolution information, particularly in remote mountainous areas [31]; thus, remote sensing constitutes an alternative approach to monitoring the dynamics of drought.

Obviously, the use of long-term remote sensing data can be employed to analyze the spatial and temporal drought characteristics in KRB, and the results of such analyses should be helpful for better understanding the local eco-environmental assessments and adaptation. To fill this gap and generally investigate the drought dynamics, the CWSI based on MOD16 product was used to evaluate the drought in KRB, and the objectives of this study are to (1) identify spatio-temporal pattern of drought, (2) to ascertain influencing factors resulting in difference in spatiotemporal distribution in KRB. This work will provide a scientific basis for the planning of water resource use, drought disaster prevention, and mitigation in KRB.

## 2. Materials and Methods

### 2.1. Study Area

**Figure 1:**
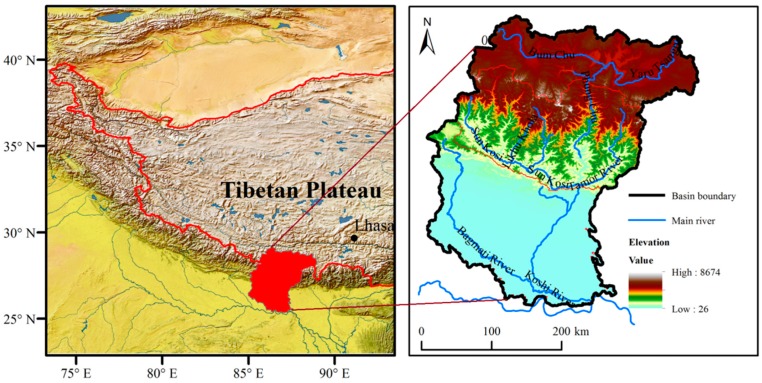
Location of the Koshi River Basin (KRB), its elevation ranges and main rivers.

Koshi River Basin (KRB) (Figure 1) (26°47~29°12’ N and 85°22’~88°21’ E) is located on the southern margin of the Tibetan Plateau, with an area of 69,300 km^2^, 32% in China, 45% in Nepal and 23% in India. Due to variations in elevation and topography, the basin is broadly divided into five parts from South to North: the Terai Plains (<500 m), the Low River Valleys (<700 m), the Siwalik Hills (700–1500 m), the Mountains (1500–2700 m), the High Mountains (2700–4000 m) and the Himalayas (>4000 m) [32]. The Koshi River Basin is mainly influenced by the South Asian monsoon during summer, which contributes to 80% of total annual rainfall [33]. Varying climate and topography results in the unevenly distributed precipitation, from 1755 mm in the central mountain to 210 mm in the trans-mountain region annually [34]. KRB has four seasons: pre-monsoon (March–May), monsoon (June–September), post-monsoon (October–November) and winter (December–February) [35,36].

The KRB has seven major sub-basins: the Tama Koshi, Arun, Dudh Koshi, Likhu, Tama, Sun Koshi, and Indrawati, and the flow directions of main rivers are from north to south due to the high elevation in the north and low in the south. Large elevation gradient and climate changes results in an obvious vertical zonation in vegetation across KRB. The vegetation across the KRB ranges from glacier, grassland and shrubland to forest and includes most of the complete vegetation types on the earth [37]. The basin has a total population of 39.2 million people, and is heavily populated in the plains and middle mountains, and scattered in the higher mountains [38], where agriculture and livestock are the main livelihood options for communities. Additionally, water is taken as a scarce resource in KRB as its availability is decreasing day by day since water is taken as a vital resource, not only for drinking purposes but also for irrigation, electricity generation and much more. Based on the landform characteristics, KRB can be divided into the downstream, midstream and upstream reaches, and most of the upstream reach is mainly distributed in China.

### 2.2. Data

The data used in this study included remote sensing data and meteorological data. The remote sensing data are the standard MODIS products provided by NASA, and the data is composed of surface Evapotranspiration (ET), Potential Evapotranspiration (PET), latent heat flux (LE) and potential latent heat flux (PLE), which is characterized by high precision, high spatiotemporal resolution and free access [26,39]. The ET and PET in this study were obtained from monthly MOD16 A2 and yearly MOD16 A3 products, with spatial resolution of 1000 arcsec (http://www.ntsg.umt.edu/project/mod16), and time range varying from 2000 to 2014 [40].

### 2.3. Crop Water Stress Index (CWSI)

Evapotranspiration is composed of soil evaporation and vegetation evapotranspiration [26]. Soil moisture supply is a fundamental process of evapotranspiration, and soil moisture (SM) exerts a huge impact on evapotranspiration. In general, when SM is sufficient, a relatively strong evapotranspiration effect occurs; on the contrary, when the SM is insufficient, the evapotranspiration effect will be relatively weak and the temperature of the vegetation canopy will be relatively high [26]. Potential evapotranspiration (PET) and Actual evapotranspiration (ET) are regarded as common indices to characterize SM. The ratio between actual ET and PET is taken as the scale for the CWSI. In addition, a series of meteorological elements (e.g., the state of vegetation cover on the underlying surface, surface wind speed and vapor pressure) were taken fully into consideration in the CWSI. Besides, the CWSI is characterized by clear physical significance and wide application range, and has been widely applied to monitor drought originated from soil moisture and farmland evaporation. The calculation of CWSI [23,41] is:

(1) $$\mathrm{CWSI}=1-\frac{\mathrm{ET}}{\mathrm{PET}}$$

The value of the CWSI ranges from 0 to 1, and the drought level can be divided into 4 types: slight drought, 0~0.25; moderate drought, 0.25~0.5; severe drought, 0.50~0.75; extreme drought, 0.75~1.0 [42].

## 3. Results

### 3.1. Test of Drought Monitoring Results

To ensure the reliability of RS-based monitoring results, the data of CWSI based on the MOD16 products was validated compared with meteorological parameters. Table 1 showed the comparison between different climatic characteristics and CWSI. It was observed that the CWSI increased with the increase in Potential evapotranspiration, and the variation of Dryness was in line with CWSI. Obviously, the higher Potential evapotranspiration and Dryness was consistent with the higher CWSI, suggesting that the CWSI can be act as an indicator to reflect the characteristics of the drought.

### 3.2. Spatial Pattern of Drought

**Figure 2:**
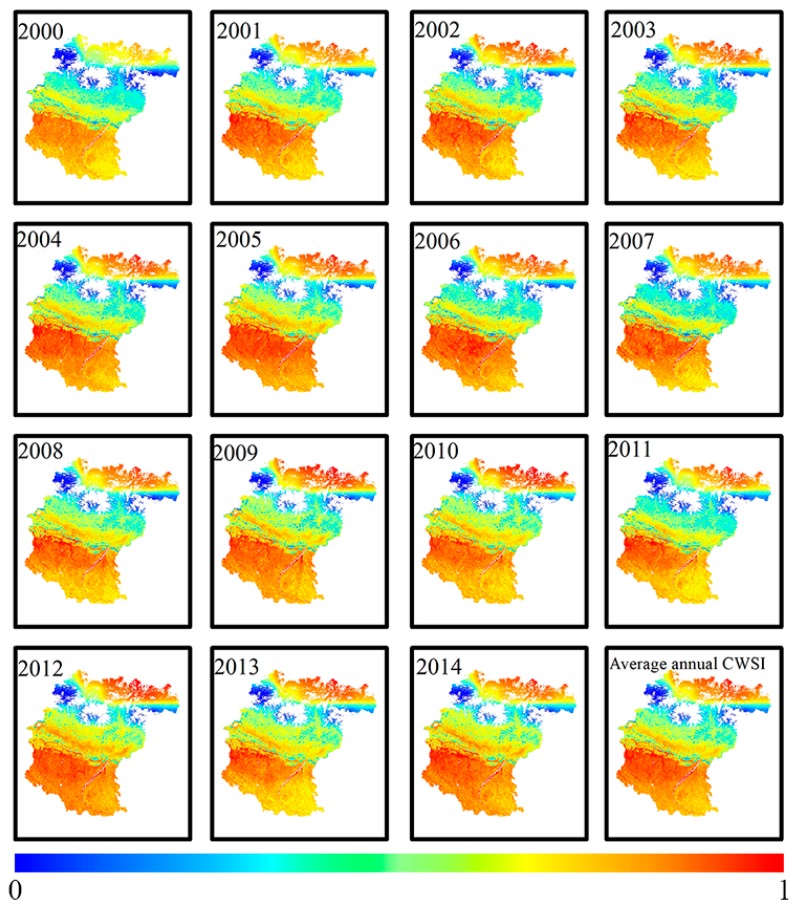
Spatial distribution of Drought degree in Koshi River Basin (KRB) in different years.

Figure 2 showed the results of spatial distribution of mean drought condition for 15 years in the KRB. Obviously, the CWSI exhibited a similar spatial distribution of drought for different years. The midstream of the KRB was dominated by light drought, moderate drought occurred in the upstream of the KRB, and the downstream was characterized by severe drought. Mean CWSI in KRB was illustrated in Figure 2. Mean CWSI range from 0.31 to 0.93 with an average of 0.76 for the downstream, from 0.23 to 0.87 with an average of 0.65 for the upstream, and from 0.23 to 0.81 with an average of 0.57 for the midstream. Obviously, these results of spatial distribution indicated that the drought followed the order: the downstream > the upstream > the midstream.

**Figure 3:**
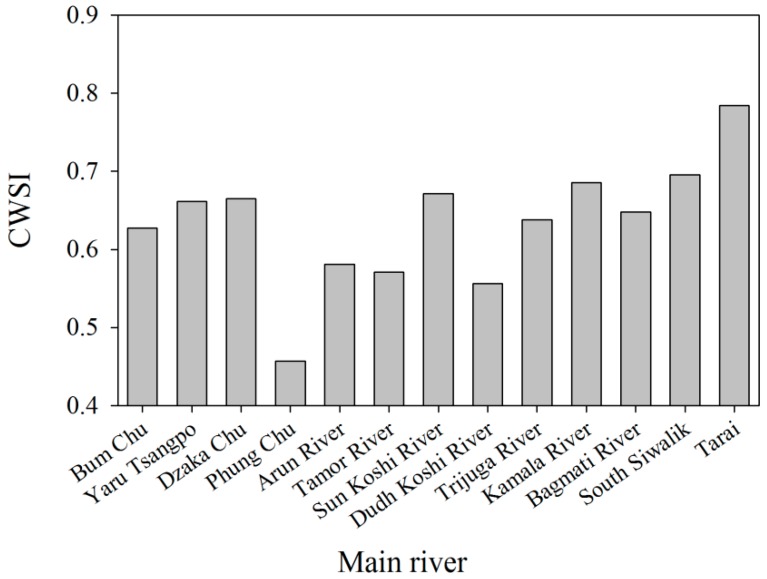
Drought degree in main rivers from Koshi River Basin (KRB).

Additionally, we analyzed the drought level of main rivers in the KRB to show drought pattern. The spatial distribution pattern of the CWSI for main rivers in the KRB for 15 years is presented in Figure 3. In upstream, the mean values from the upstream including Bum Chu, Yaru Tsangpo and Dzaka Chu ranges from 0.43 to 0.66, with an average of 0.54. However, in midstream, the mean CWSI values varied between 0.55 and 0.65, with an average of 0.59. The CWSI values in downstream are significantly higher than those of midstream and upstream, ranging from 0.62 to 0.76 with an average of 0.67, indicating that the downstream suffers extreme drought in the KRB.

### 3.3. Temporal and Spatial Variation in Drought

**Figure 4:**
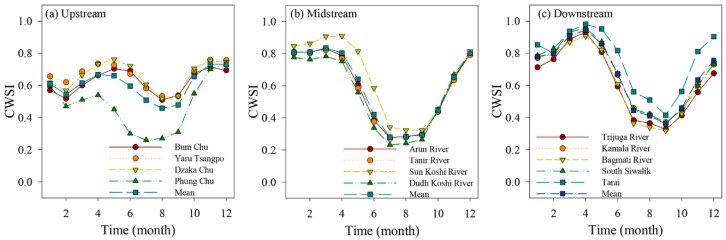
Annual distribution of Drought degree in Koshi River Basin (KRB).

The temporal pattern of drought was conducted based on month, seasonal and annual variation. In upstream, the variation trend is similar for different rivers, and can be divided into four stages (Figure 4). The CWSI mainly included gradually increasing trend (February to May), decreasing trend (May to August), significantly growing trend (August to November) and gradually decreasing trend (November to February in the next year). In contrast to four stages in upstream, the midstream is characterized by higher-stable (December to April) and lower-stable (July to September) CWSI values. However, the downstream exhibited a different distribution pattern of CWSI. From April to September, the CWSI exhibited a remarkably decreasing trend and a significantly growing trend. A significantly growing trend was observed between September and April in the next year. Additionally, lower CWSI mainly occurred between June and October in overall KRB. From the mean CWSI values from different rivers in KRB, the ranking order was the midstream < the upstream < the downstream.

**Figure 5:**
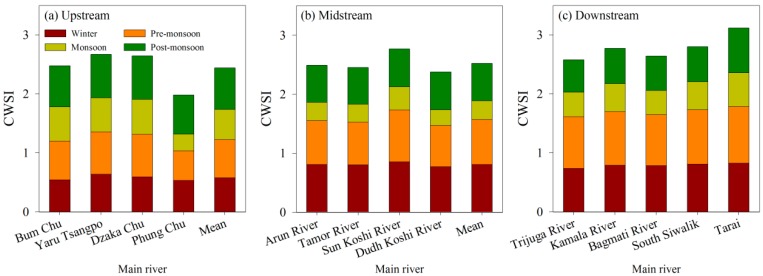
Distribution of Drought degree from different monsoon stages in Koshi River Basin (KRB).

Figure 5 shows the distribution of CWSI from four seasons, winter, pre-monsoon, monsoon and post-monsoon. Obviously, the mean CWSI from different rivers showed a similar pattern, and the lowest CWSI occurred in Monsoon period. For pre-monsoon, the CWSI in downstream was significantly higher than that of the midstream and that of the upstream, indicating that severe drought occurred in the downstream and light drought occurred during the pre-monsoon. For winter and pre-monsoon, the light drought occurred in upstream; while the monsoon and post-monsoon showed light drought in the midstream, suggesting that significant drought occurred in the pre-monsoon.

**Figure 6:**
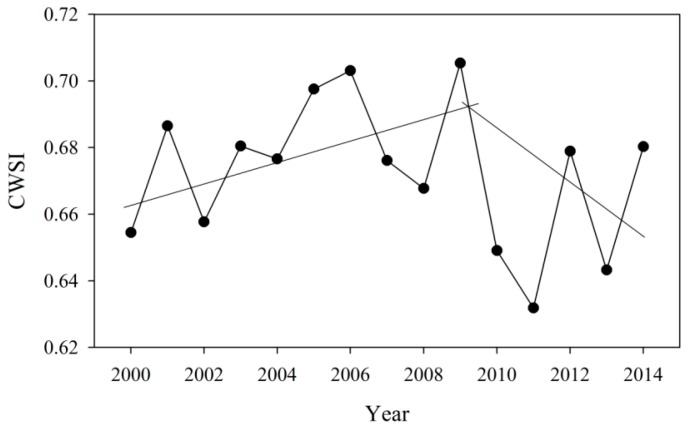
Annual variations of CWSI of KRB during 2000–2014.

Figure 6 presents annual variations of CWSI of KRB during 2000–2014, and the CWSI value ranges from 0.63 to 0.71, indicating that overall the Koshi River Basin is dominated by moderate drought. The CWSI ranging from 2000 to 2014 shows significant interannual fluctuation, and higher CWSI occurred in 2001, 2005, 2006 and 2009, with the lower values in 2010, 2011 and 2013. For 15 years CWSI, we could divide into two stages: rising stage and falling stage. During 2000 to 2009, the CWSI showed an obvious increasing trend, suggesting that the drought degree increases. However, a significant fluctuant falling trend in CWSI was observed from 2009 to 2014.

## 4. Discussion

The spatial variability of drought based on the CWSI was investigated from 2000 to 2014. Our results showed that the drought degree in midstream was significantly lower than that of the upstream and downstream. In addition, slight drought occurred in the monsoon, the most serious drought was observed during the pre-monsoon. Compared with the midstream and the downstream, the fluctuation of drought was relatively lower in upstream.

**Figure 7:**
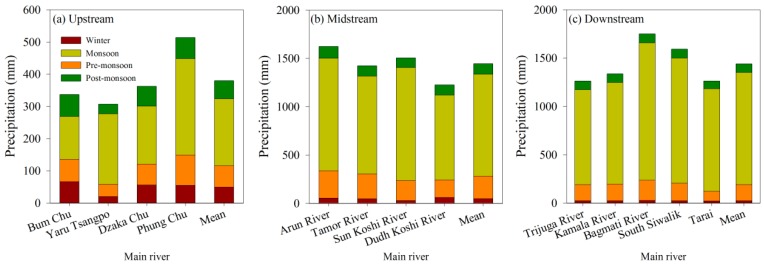
Precipitation from different rivers in Koshi River Basin (KRB).

**Figure 8:**
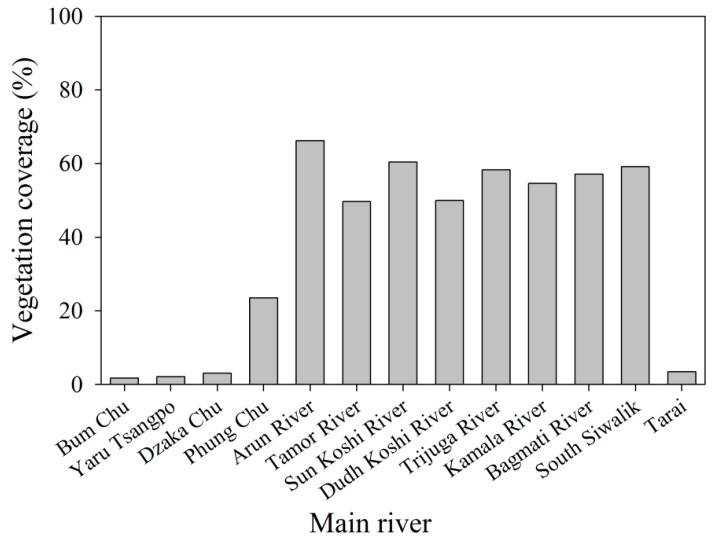
Vegetation coverage from different rivers in the Koshi River Basin (KRB).

Significant difference in drought in KRB was observed in this study, and the rank of the drought order was the midstream < the upstream < the downstream. The results were consistent with the study of Dutta et al. (2015) that the midstream of the objective area was lower than the upstream and the downstream [11]. This is partially attributed to the overall effects of precipitation, temperature, vegetation cover and water conservancy facilities. In general, the temperature declines from South Teri Plains to North High Mountains due to the altitudinal effects [43,44]. Precipitation in the KRB increases from the Low River Valleys to the Mountains and then decreases in regions of higher elevation like the High Mountains and Himalayas [45]. Although the precipitation follows the order: the midstream > the downstream > the upstream (Figure 7), the evaporation is the downstream > the midstream > the upstream, i.e., higher temperature in downstream caused the evaporation variation, leading to the sequent higher evaporation. Besides, approximately 60% of vegetation coverage occurred in Midstream area (Figure 8), which results in the reduction in drought degree. The area of forest coverage in midstream in KRB is significantly higher than that of the upstream and downstream [34,46]. In addition, the lack of water conservancy facilities also exacerbated the drought degree to some extent in the downstream. According to our field observation in 2017, scarce water facilities were observed in the downstream, accordingly resulting in the reduction in storage water capacity. Even if higher precipitation occurred during the monsoon, the lower storage water capacity and higher evaporation aggravated the drought degree in downstream of the KRB.

Based on the characteristic of the southwest monsoon, the KRB has four seasons: pre-monsoon (March–May), monsoon (June–September), post-monsoon (October–November) and winter (December–February) [36,47]. Due to the uneven precipitation, the climate is characterized by a distinct dry-wet season [47]. The specific climate characteristic would be expected to have a significant impact on drought. Our study showed that the CWSI in monsoons was significantly lower than that of other seasons, including the winter, pre-monsoon and post monsoon. Similar results were also observed by Pen et al. (2009) in that a relatively light drought occurred in June to September in Yunnan Province, Southwest China [48]. Therefore, it can be inferred that higher precipitation during the monsoon significantly decreased the overall drought degree. Southeasterly monsoon originating from India ocean dominates overall climate of KRB, providing 80% of annual precipitation during summer months from June to September [32] (Figure 5). A large amount of precipitation effectively increases soil moisture, and reduces the agricultural drought during the monsoon.

Besides, our results also showed an obvious spatial difference of drought for the same period. For Cold season and Pre-monsoon, the CWSI rank of the order was the upstream < the midstream < the downstream, indicating that the degree of drought decreased with the increasing elevation. The lower temperature reduced evaporation, resulting in lower drought compared to the midstream and upstream. For the monsoon and post-monsoon, the CWSI ranking order was the midstream < the upstream < the downstream. Due to the decreasing temperature and precipitation [32], the results were related to the fact that the relatively high water conservation function occurred in midstream. The distribution pattern of forest cover degree has a significant impact on drought in the KRB. The forest coverage rate in midstream is relatively high, with a percent of 50% from many rivers, such as the Arun river, Sun Koshi river and Dudh Koshi river (Figure 8). However, the sub-rivers located on the north slope of the Himalayas and Terai plain showed a relatively lower forest coverage rate with a percentage of 5%. Higher forest coverage can moderate surface flow, cut flood peak, playing an important role in reducing flood disaster and drought. Our results also demonstrated that the most serious drought occurred in the pre-monsoon, indicating that long periods of no precipitation and high evaporation aggravated the drought degree, which is a threat to agricultural activities. In Nepal, 65% of arable land is rain-fed, with only 24% with access to irrigation systems [49]. Most of the irrigable land is in the Terai; there is some irrigation in the middle hills and mountains, primarily limited to small-scale surface irrigation and micro-irrigation system, such as drip or limited sprinkler systems, which could not ease the damage to drought severity.

Finally, our results demonstrated that the most serious drought occurred in downstream of KRB, and the necessary water conservancy facilities should be constructed to mitigate the drought. The lighter drought was found in the monsoon, suggesting that precipitation can reduce drought degree in Monsoon. For annual variation of drought, the most serious drought existed in the pre-monsoon, and the local farmers should take measures to adapt agricultural practices and storage water, to reduce drought loss in KRB.

## 5. Conclusions

In this study, drought pattern was assessed using MODIS products based on CWSI for the period from 2000–2014. The results demonstrated that drought is one of the climate-related disasters for people’s livelihoods during the analyzed period in the KRB. A significant difference of drought pattern occurred in the KRB, and the rank was the midstream < the upstream < the downstream, suggesting that the downstream should be regarded as the main area to mitigate drought. Besides, the slight drought occurred during the monsoon due to concentrated rainfall. Significant variation of CWSI was observed in midstream and downstream, indicating that the above areas are susceptible to climate change. The most serious drought occurred during the pre-monsoon due to long-term rainfall shortage. To mitigate server drought during the pre-monsoon, some water conservancy facilities, such as pond and dam, should be constructed in downstream, and water-dependent crops should be not arranged in dry season for the pre-monsoon.

## Figures and Tables

**Table 1:** Climatic characteristics of the KRB.

ID	Precipitation (mm)	Temperature (°C)	Potential Evapotranspiration (mm)	Dryness	CWSI
Upstream	658.8	3.8	739.2	1.19	0.65
Midstream	1373.1	13.9	864	0.6	0.57
Downstream	1137.73	25.48	1616.6	1.49	0.76
